# Online Self-Management Support for Family Caregivers to Help Them Manage Behavior Changes in Their Relative With Dementia: Study Protocol for a Randomized Controlled Trial and a Process Evaluation

**DOI:** 10.2196/resprot.8365

**Published:** 2017-11-28

**Authors:** Judith G Huis in het Veld, Bernadette M Willemse, Iris FM van Asch, Rob BM Groot Zwaaftink, Paul-Jeroen Verkade, Nienke J Veldhuijzen, Renate Verkaik, Marco M Blom, Anneke L Francke

**Affiliations:** ^1^ Department of Public and Occupational Health VU University Medical Center Amsterdam Netherlands; ^2^ Trimbos Instituut Netherlands Institute of Mental Health and Addiction Utrecht Netherlands; ^3^ Dutch Alzheimer’s Society Amersfoort Netherlands; ^4^ The Geriant Foundation Region North of Amsterdam Netherlands; ^5^ Amsterdam Public Health Research Institute VU University Medical Center Amsterdam Netherlands; ^6^ Netherlands Institute for Health Services Research Utrecht Netherlands

**Keywords:** dementia, family caregivers, self-management, behavior problems, Internet, eHealth, RCT, process evaluation

## Abstract

**Background:**

Online interventions are potentially effective ways to support family caregivers in the management of behavior changes in their relative with dementia.

**Objective:**

The objective of this paper is to present the design of a study evaluating and comparing 3 intervention arms for online self-management support.

**Methods:**

A randomized controlled trial (RCT) will be conducted with a total of 81 family caregivers of community-dwelling people with dementia in the Netherlands. Family caregivers will be randomly allocated to one of the following intervention arms: (1) a major self-management support intervention consisting of personal email contacts with a nurse specialized in dementia care, online videos, and electronic bulletins (e-bulletins); (2) a medium self-management support intervention consisting of only online videos and e-bulletins; and (3) a minor self-management support intervention with only e-bulletins. The primary outcome is the self-efficacy of the family caregiver. The secondary outcomes are the behavior problems of the person with dementia as reported by the family caregiver, and positive and negative aspects of the relationship. Background characteristics (eg, type of family relationship) will also be assessed. All data for the RCT will be collected via online questionnaires, administered before the intervention (T0), after 6 weeks (T1), and after 12 weeks (T2). Alongside the RCT, a process evaluation will be conducted, based on a number of evaluation questions and semi-open interviews with family caregivers.

**Results:**

Data collection will be completed in August 2017. Study results will be reported in early 2018.

**Conclusions:**

The study will shed more light on the effect of online self-management support interventions and insights will be gained into whether a major intervention, consisting of personal email contacts with specialized nurses, videos, and e-bulletins, has more effect than smaller online interventions. This is relevant in an age with increasing numbers of people with dementia, growing pressure on family caregivers, more and more people using the Internet, and increasing healthcare costs.

**Trial Registration:**

Nederlands Trial Registry (NTR): NTR6237; http://www.trialregister.nl/trialreg/admin/rctview.asp?TC=6237 (Archived by WebCite at http://www.webcitation.org/6v0S4fxTC)

## Introduction

### Background

Dementia is a progressive disorder characterized by cognitive and physical decline and behavior and mood changes. The most common forms of dementia are Alzheimer's disease and vascular dementia, followed by Lewy body dementia and frontotemporal dementia [1]. There is still no effective treatment that can influence the progression of Alzheimer’s disease and other dementia subtypes. Eventually, someone will die with or from dementia [2].

Most people with dementia live at home, often supported by spouses, adult children, or other family members [3]. Although the family often cares for them with love and dedication, family care can be a big burden [4,5]. For family caregivers it can, for instance, be stressful to deal with their relative’s behavior changes, such as dependent behavior, aggressive behavior, suspicious behavior, apathy or indifference, night-time restlessness, and masking behavior. These are often symptoms of the disease and are found in up to 90% of people with dementia [6,7]. Changes in behavior are “challenging” when this causes distress to the person with dementia and/or family caregivers and negatively affects the quality of life of at least one of these parties [8]. A Dutch study [9] found that about three quarters of the family caregivers of persons with dementia experienced problems in dealing with changes in the behavior or mood of their relative, in both the initial and later stages of the disease. In a recent focus group study, family caregivers reported that what they found most difficult was constantly having to switch between different strategies and that they had to keep their relative constantly occupied and distracted [10]. Furthermore, they found it stressful that other people often had a different view of the behavior and mood of the relative with dementia. Lastly, they also found it difficult that in theory they knew what to do in caring for their relative, but were often not able to put it into practice [10].

To support family caregivers (eg, in dealing with the relative’s behavior changes), an increasing number of self-management support interventions are being developed, some of which are Internet-based [11]. From the perspective of family caregivers, Internet support might be attractive, since they can use it at a time that is suitable for them, without travelling [12]. Boots et al [13] performed a systematic literature study of Internet-based support, such as a website with information and support on various aspects of care giving. The review by Boots et al [13] suggested that Internet-based support had positive effects (eg, regarding self-efficacy or other psychological and psychosocial outcomes for family caregivers). However, the review authors also concluded that the evidence was still scarce because of the low quality of the studies they had identified [13].

Previous research also did not provide a definitive answer about the effectiveness of incorporating personal contacts with a healthcare professional in online interventions, although some relevant studies have been conducted in this area [12,14-16]. For instance, in a study by Boots et al [14], face-to-face sessions with an experienced professional (psychologist or psychiatric nurse) and family caregivers were added to an online support intervention. The face-to-face sessions were seen as a valuable addition, as they provided an opportunity to tailor the support to the needs of the family caregivers and deepened the relationship [14]. In addition, Schaller et al [15] evaluated an interactive Web portal providing individualized information and support by dementia experts to family caregivers via a messaging function [17]. The interaction between family caregivers and experts was found to be useful, particularly because of the timely reaction to symptoms and because of the opportunity to reach immobile caregivers [15]. Comparable results were found in the study by Torkamani et al [16], which evaluated a computerized platform for contacts between the caregivers and health professionals aimed at reducing the burden on the caregiver, improving quality of life, and delaying institutionalization of the person with dementia. Furthermore, Blom et al [12] evaluated Internet-based information combined with online personal support. In this study, a psychologist provided online feedback on assignments about dealing with depression or other psychological problems in relatives of persons with dementia. The study by Blom et al [12] recommended further research to clarify the necessity of personal contacts with a professional; a completely self-help Internet program would be less expensive, which is an advantage in the current era with increasing numbers of persons with dementia and limited healthcare budgets. However, personal contacts with a healthcare professional might help people translate generic information to their own situation [12].

### Objectives

The aim of this study is to investigate whether a major intervention, consisting of personal email contacts with a specialized nurse in combination with videos and electronic bulletins (e-bulletins), is more effective than more minor interventions. Based on the results of this study we will be able to inform about which elements of online self-management support are effective (on their own or in combination) for family caregivers when managing changes in the behavior of their relative with dementia.

The research questions are (1) Is a major online self-management support intervention consisting of personal email contacts with a specialized dementia care nurse, videos, and e-bulletins more effective than smaller online interventions without personal email contacts, with regard to self-efficacy of family caregivers in managing the behavior changes of their relative with dementia, behavior problems in the persons with dementia, as reported by family caregivers, and positive and negative aspects of the relationship between the family caregiver and the person with dementia? (2) What background and baseline characteristics of family caregivers or the persons with dementia (eg, type of family relationship, baseline level of care pressure, and the specific behavior problems of the person with dementia) are associated with effects on the outcome variables mentioned in question 1? (3) How do the family caregivers evaluate the online self-management support intervention, with or without personal email contacts with a specialized nurse, regarding feasibility, usability, and satisfaction with the intervention?

## Methods

### Design and Randomization

To answer research questions 1 and 2, a randomized controlled trial (RCT) with 3 repeated measurements will be performed, involving the following intervention arms: (1) a major-intervention arm, (2) a medium-intervention arm, and (3) a minor-intervention arm.

Family caregivers will be randomly allocated to 1 of the 3 self-management intervention arms. Block randomization will be used to achieve balance in the allocation of participants to intervention arms [18]. An independent epidemiologist (NJV) prepared a randomization schedule to assign participants to an intervention arm, using several block sizes of 6 and 9. Following this randomization schedule, the researcher (JGH) will allocate participants to an intervention arm. The participants will then receive an email from the researcher (JGH) containing elements of the intervention arm in question. Participant and researcher blinding is not possible due to the nature of the intervention arms and the organization of the study.

Alongside the RCT, a process evaluation will be conducted to answer research question 3. For the process evaluation, a mix of qualitative and quantitative methods will be used.

### Power Calculation and Sample

We hypothesize that (1) both the major and medium intervention arms improve the self-efficacy as compared to the minor intervention arm; and (2) the major intervention arm gives better results for self-efficacy compared to the medium intervention arm. Considering a difference of 0.8 standard deviation units between the groups and assuming a significance level of .05, a power of 80%, and a correlation of .6 between the 2 repeated measures, 20 participants are needed per group. Taking into account a drop out percentage of 20%, we will include 24 participants per group.

In this study, providing self-management support through email is a relatively new task for the specialized nurses involved, with possible learning effects during the study. To take this into account, 1 extra block of 9 participants will be added to allow for a brief learning curve. Hence, in total 81 family caregivers of persons with dementia will be included.

The participants will be family caregivers of people with dementia who meet the following inclusion criteria: (1) the family caregiver is a relative of a person diagnosed with dementia (all types of dementia are eligible, with no restriction on the severity of the dementia); (2) the family caregiver must have contact with the person with dementia at least once a week; (3) the family caregiver's relative with dementia has to live at home (not in a care institution); (4) the family caregiver has access to the Internet and has basic skills in using the Internet and email; (5) the family caregiver has to be aged at least 18 years of age; and (6) the family caregiver is able to read and write Dutch.

To recruit family caregivers for our study, we will use several channels. The panel of the Dutch Alzheimer Society (in which more than 3000 informal caregivers participate) will be sent an email with an open call. Open calls will also be posted on the online forum of the Dutch Alzheimer Society (with 7000 monthly visitors), on the Dementie Nederlands website, and on the social media accounts (Facebook/Twitter) of the Dutch Alzheimer Society.

**Figure 1:**
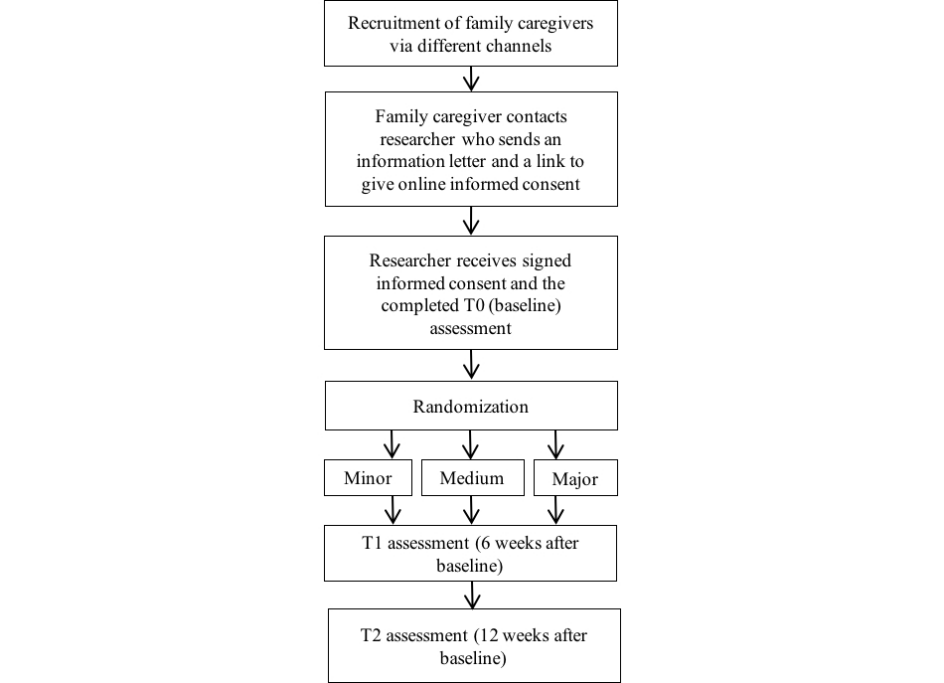
Study flow chart.

Recruitment via the aforementioned channels of family caregivers will proceed with first, a very short study description in the open call. In this description, family caregivers will be asked if they are interested in participating in the study. If so, they can send their name and email address to the principal researcher (JGH). The principal researcher will then send an email containing an information letter about the aims and procedures of the study to the family caregiver. This email will have a link to an online informed consent form, which the family caregiver can use to give their consent for participation. The participation flow chart is shown in Figure 1.

### Intervention Arms and Components

In the RCT, 3 intervention arms will be studied, all focusing on self-management support in dealing with behavior changes, but varying in the number of elements. The intervention arms are referred to as major, medium, and minor.

#### Major Self-Management Support Intervention

The major intervention arm consists of the following elements: (1) 3 personal email contacts with a nurse specialized in dementia care, (2) provision of online videos about how to manage behavior changes in a relative with dementia and to improve your self-efficacy in managing with this behavior, and (3) provision of e-bulletins with practical information about different types of behavior changes and how to manage them.

The personal email contacts will be handled by a nurse with a Bachelor’s or Master’s qualification in nursing and with follow-up training in dementia care. In the email contact, the nurse will support the family caregiver in managing behavior changes. The nurse will also give feedback on assignments and will give feedback on the plan that the family caregiver came up with in the assignments. The nurse will tailor their support to the personal needs and questions of the family caregiver, while guided by an intervention protocol developed by project group members (JGH, ALF, PJV, IvA), in consultation with the nurses who had to use the intervention protocol. The number of email contacts was discussed and agreed with experts in dementia care who have experience with online support. Three email contacts are thought to be sufficient and feasible.

The Dutch-language intervention protocol (available on request from the first author) is based on the 5 steps of the “5A model” of self-management support [19] and the person-centered care theory of Kitwood [20]. The “5A model” consists of the following steps: (1) assessing the state of behavior, beliefs, and motivation; (2) advising based upon personal health risks; (3) agreeing on a realistic set of goals; (4) assisting in anticipating barriers and developing a specific action plan; and (5) arranging follow-up [19,21,22].

There are 6 videos about different types of behavior changes that occur frequently (dependent behavior, aggressive behavior, suspicious behavior, apathy or indifference, nighttime restlessness, and masking behavior). Family caregivers can choose the number of videos they watch and the accompanying assignments that they do themselves, depending on their own needs and the behavior changes that occur in their relative with dementia. The videos (as well as the e-bulletins mentioned below) were developed by the Trimbos Institute, of which 2 of the developers are involved in the present study (BMW, IvA), in close cooperation with the Dutch Alzheimer’s society, other dementia experts, and family caregivers of people with dementia. As a first step in the development trajectory, a desk search was performed to gain insight into what is known in the literature about how family caregivers perceive different types of behavior changes in their relative with dementia and the theory of person-centered care [20]. Experts also provided input for the components of the videos (eg, principles of cognitive behavioral therapy [CBT], modeling, persuasive communication, and active learning). At several stages in the development trajectory, video scripts and pilot videos were tested by family caregivers.

The behavior changes covered in the bulletins are the same as in the videos. The e-bulletins involve assignments to help caregivers translate the generic information to their own situation and to reflect on possible causes of the behavior changes, how they want to influence the behavior, and how they want to cope with it. The e-bulletins were tested in conjunction with the testing of the videos and they also have the same theoretical base as the videos.

#### Medium Self-Management Support Intervention

The medium self-management support intervention consists only of the online videos and e-bulletins as described above.

#### Minor Self-Management Support Intervention

The minor self-management support intervention consists only of the e-bulletins, the same as those in the major and medium support interventions.

### Measurement Procedures

Measurements will be performed in the RCT at 3 time points: baseline assessment (T0), which is just before the family care intervention arms start; the assessment 6 weeks after the baseline (T1); and the assessment 12 weeks after the baseline (T2). Measurements will be based on self-reporting by the family caregiver and will be administered through the Internet. Up to 2 email reminders will be sent (if necessary) 1 and 2 weeks after the measurement time point to remind participants to complete the questionnaires.

### Primary Outcome

The primary outcome in the RCT is self-efficacy, measured by the Trust in Own Abilities (TOA) instrument, a Dutch language questionnaire to be completed by family caregivers of the person with dementia [23]. The questionnaire has been used before to measure self-efficacy in caregivers of people with dementia living at home [24]. The TOA contains 32 items (alpha .97) divided into 3 subscales: resilience (15 items, alpha .94), solution orientation (8 items, alpha .90), and proactive competence (9 items, alpha .81). Items are measured on a 5-point Likert scale, ranging from 0 (not at all) to 4 (very good). A higher score is associated with higher perceived competence in taking care of the person with dementia [24].

### Secondary Outcomes

The secondary outcome will be the presence and number of behavior and mood problems, assessed with the Dutch version of the Revised Memory and Behavioral Problem Checklist (RMBPC) [25,26]. Family caregivers have to rate the frequency of the occurrence of a specific behavior or mood problem on a scale from 0 (never) to 4 (always) where 1 is seldom, 2 regularly, and 3 is often. The total number of behavior and mood problems (0 to 24) will be calculated as well as the mean overall score. The RMBPC can be divided into scales for depression (9 items), disruptive behavior (8 items), and memory-related problems (7 items).

Another secondary outcome is the positive and negative aspects of the family relationship between the family caregiver and the person with dementia and they will be measured by the Dyadic Relationship Scale (DRS). The family caregiver version includes 11 items in 2 subscales: dyadic strain and positive dyadic interaction. Family caregivers have to rate the quality of the relationship using 4 answer categories: 1 (strongly disagree), 2 (disagree), 3 (agree), and 4 (strongly agree) [27].

### Analyses of Effects

The quantitative data from the RCT will be analyzed using SPSS software (Statistics 22). Baseline characteristics will be described for each arm using proportions for dichotomous variables and means (SD) or medians (IQR) for continuous variables. In the primary analysis, primary and secondary outcomes will be compared between the 3 different groups using mixed-models analysis. All mixed model analysis will be adjusted for baseline differences between the groups.

All randomized caregivers who completed the follow-up will be included in this analysis (modified intention-to-treat). The first 9 caregivers, who are in the learning-curve block, will not be included in the primary analysis. We will use sensitivity analyses to evaluate the effect of missing data and of the prior inclusion of key baseline variables.

### Process Evaluation

Alongside the RCT, a process evaluation will be conducted. Mixed-methods and sources will be used for this. Firstly, evaluation questions will be included in the T2 survey questionnaire (12 weeks after the baseline). The number of evaluation questions varies between 5 and 11 depending on which of the 3 intervention arms the family caregiver is in. The evaluation questions are based on earlier research about the perceived feasibility and usability of interventions and satisfaction with the interventions [28,29].

Secondly, semi-structured interviews will be conducted with a purposive sample of about 15 participant family caregivers (5 participants in each intervention arm). The participants will be purposively recruited to achieve a spread in the intervention arms and background characteristics (eg, sex, age, and living with or separately from the relative with dementia). Topics will include family caregivers' satisfaction with and the perceived feasibility and usability of the self-management support interventions. The interviews will be conducted by telephone by 1 of the members of the research team (IvA) and will be audio-recorded.

Thirdly, usability in the sense of actual usage of the different elements of the online self-management support intervention will be measured by analyzing the clicks on links and how long the family caregivers spent watching the videos, divided into the following categories: (1) started video, (2) played video (25%), (3) played video (50%), (4) played video (75%), and (5) completed video. These data will be collected with Google Analytics. All participating family caregivers will be given a unique code that is known only by the research team.

To collect data on actual use of the personal email contacts, nurses will be asked to complete a registration form on the number of personal email contacts per family caregiver and time spent on giving feedback to the family caregiver.

Fourthly, the content of the email contacts between the family caregivers and the nurses will be analyzed qualitatively. The email contacts will be analyzed from 3 angles: with a focus on nurses’ questions and responses, with a focus on family caregivers’ questions and responses, and with a focus on the interactions between the two. The focus on the nurses will be on how they delivered the self-management support as defined by the intervention protocol based on the “5A model” (assess, advise, agree, assist, and arrange). The responses by the family caregivers in the email contacts will be analyzed to get information on the uptake of the intervention and how they integrated the personalized advice from the nurse in their daily lives.

The data from the structured evaluation questions in the T2 survey questionnaire, data on actual usage from Google Analytics, and registration data on the number of personal email contacts will be analyzed descriptively using SPSS software. The semi-open interviews and the content of the email contacts will be analyzed qualitatively using the principles of thematic analyses [30]. This qualitative method was chosen because it is a useful and flexible method for identifying relevant themes within qualitative data. It consists of the following steps: (1) familiarizing yourself with the data, (2) generating initial codes, (3) searching for themes, (4) reviewing themes, (5) defining and naming themes, and (6) reporting [30]. The interview transcripts will be analyzed by 2 researchers (JGH and IvA) independently. Coding and interpretation of the codes will be discussed by the researchers until consensus is reached. In addition, other authors will comment on the interim analyses of the interviews.

### Ethical Procedures

The study protocol was approved by the Medical Ethics Committee of the VU University Medical Center (reference 2016.559).

Informed consent will be asked from all participants via an online informed consent form, which the family caregiver can use to give consent for participation. Consent from the family caregivers and the nurses will be explicitly requested in the informed consent for the analysis of the content of the email contact between the family caregivers and the nurses.

Only members of the research team (the co-authors) will have access to the data. Agreements on how to share, archive, and store data will be signed by the organizations that will be collecting the data.

## Results

Enrollment of participants began in March 2017. Data collection was complete in August 2017. The study results will be reported in early 2018.

## Discussion

This study will contribute to the growing body of knowledge about online support in dementia care. This is important since future generations will increasingly use the Internet, which will also affect the extent in which family caregivers will be open to receiving online self-management support. However, we also expect that if online support is tailored and involves personal email contacts with a specialized nurse, this will be more effective and more satisfying for the family caregiver than if only online videos or e-bulletins are provided. The study results will be used to inform care professionals and family caregivers about which forms of online support intervention are most effective and best match family caregivers' needs.

## Abbreviations

DRS: Dyadic Relationship Scale
e-bulletin: electronic bulletin
RCT: randomized controlled trial
RMBPC: Revised Memory and Behavioral Problem Checklist
TOA: Trust in Own Abilities
